# Supplementary material for: Flanking heterozygosity influences the relative probability of different base substitutions in humans
Source: R Soc Open Sci. 2019 Sep 25;6(9):191018. doi: 10.1098/rsos.191018 (PMC6774961; doi:10.1098/rsos.191018)
Supplement: Annotated C++ code used for analysis [file rsos191018supp1.docx]

// Example code used to parse and analyse the 1000 unzipped combined, single chromosome vcf files. I // am not a programmer, as will become obvious. This code should run if pasted into a source window in, // for example, Visual Studio 2015 but you will need three additional files: (1) a 1000 genome

// individual chromosome .vcf file, renamed “ALL.chr1.vcf” (for chrom 1); (2) a list of the 2504

// samples named “inpops.txt”, a tab delimited file in which each row contains 4+ columns of which

// column 2 contains a number from 1-26 indicating population (1=GBR, 2=FIN etc.) and column 3

// contains a number between 1and 5 indicating major geographic region (1=Europe, 2=East Asia etc.);

// (3) the human reference sequence as used by the 1000 genomes project, hs37d5.fa. I am more than

// happy to help try to sort issues if they arise but I suspect most competent C-programmers will

// prefer to recode properly.

// I’m afraid I am lazy and tend to include a number of standard headers regardless of whether they

// are needed in the current code

#include <iostream>

#include <stdlib.h>

#include <fstream>

#include <string.h>

#include <stdio.h>

#include <math.h>

using namespace std;

void LoadReference(int chrom);

void LoadPops();

void Load_First(ifstream& in);

void Increment(ifstream& in, int h);

int cng(char base);

char convert(char base);

int triplet(int b1, int b2, int b3, int b4);

char line[2000000]; // array for taking possibly large data chunks from vcf file

int REF[250000000]{}; // reference sequence

char alleles[100000][2]{}; // alleles

int pops[2505][2]{}; // population data

float het[100000][10]{}; // SNP data including heterozygosity, analysed as a ‘circular’ array

long location[100000]{}; // SNP locations

int counts[30][128][5][5][2] {}; // counts of triplets[het][trip][pop][nvariants][tv/ts]

int chrom = 0;

int window = 1000; // size of window to be analysed

int main()

{

## input the chromosome to analyse and convert to a number

char chromosome[5];

cout << "chromosome ";

cin >> chromosome;

if (chromosome[1] == '\0') chrom = chromosome[0] - 48;

else chrom = (chromosome[0] - 48) * 10 + chromosome[1] - 48;

## open the appropriate vcf file, (on my computer stored on drive B)

char infile3[200] = "B:\\All.chr";

strcat_s(infile3, chromosome);

strcat_s(infile3, ".vcf");

ifstream in3(infile3);

if (!in3) cout << "input file 'ALL.chr' not open, does it exist?";

## scroll through the header lines

for (int i = 0; i < 253; i++) in3.getline(line, 100000);

## load triplet conversion array, population data and reference sequence

LoadPops();

LoadReference(chrom);

cout << "loaded\n";

## create and open the output file

char outfile[200] = "F:\\Triplet_New_W1Kn_CHR";

strcat_s(outfile, chromosome);

strcat_s(outfile, ".txt");

ofstream out(outfile);

if (out) cout << "out file open\n";

## zero loop variables and load the first 100000 SNPs

bool test = true;

int start = 0;

int lcn = 0, mb = 0, prev = 0;

Load_First(in3);

## analyse the chromosome file, terminating when it finds a location of -1 (test = True)

while (!in3.eof() & test) {

if (het[lcn][7] > -1) { // if the data array has an allele stored

float tothet = 0;

int pop = het[lcn][6];

// set start place and scroll through array, summing heterozygosity for sites

// within ‘window’ bases of the focal SNP. If ‘start’ is more than ‘window’

// bases before the focal SNP, use ‘increment’ to read more data.

int g = start;

while (location[g] < (location[lcn] + window) & test) {

if (abs(location[g] - location[lcn]) < window) tothet += het[g][pop + 1];

if (location[g] < (location[lcn] - window)) { // move forwards

Increment(in3, g);

start = g+1;

// I use a ‘circular’ array, returning to zero it reaches 10000

if (start == 10000) start = 0;

// output to screen as each megabase passes

mb = location[lcn] / 1000000;

if (mb > prev) cout << location[lcn] << "\n";

prev = mb;

}

g++;

if (g == 10000) g = 0;

// exit flag triggered if location is -1

if (location[g] < 0) {

test = false;

break;

}

}

// analyse the triplet, composed of base1,2 & 3.

// poor programming style, sorry. Integers stored in a float array may appear

// as 6.99999 instead of 7, hence the addition of a fraction when reverting

int base1 = REF[location[lcn] - 1];

int base2 = int(het[lcn][7]+0.0001);

int base3 = REF[location[lcn] + 1];

int base4 = int(het[lcn][9] + 0.0001);

int trp = triplet(base1, base2, base3, base4);

int typ = int(het[lcn][8] + 0.001) - 1;

int H = tothet*5; // spread the heterozygosity values

if (H>49) H = 49;

counts[H][trp][pop][int(het[lcn][8])-1][int(het[lcn][9])]++; // store in array

}

// counter that also uses the ‘circular’ approach, returning to zero at 10000

lcn++;

if (lcn == 100000) lcn = 0;

}

// output to file

for (int trans = 0; trans < 2; trans++) {

for (int pop = 0; pop < 5; pop++) {

for (int h = 0; h < 50; h++) {

out << h;

for (int typ = 0; typ < 5; typ++) {

for (int trp = 0; trp < 32; trp++) out << "\t" << counts[h][trp][pop][typ][trans];

}

out << "\n";

}

out << "\n";

}

}

out.close();

return 0;

}

// adds one more SNP to the circular array, for description, see ‘Load_First’ below

void Increment(ifstream& in, int h)

{

char temp[10000];

char ref[1000];

char alt[1000];

bool test = false;

long loc = 0;

while (!test & !in.eof()) {

in >> temp >> loc >> temp >> ref >> alt >> temp >> temp >> temp;

int g = 0;

bool SNP = false;

while (temp[g] != '\0' & !SNP) {

if (temp[g]=='V' & temp[g+1]=='T' & temp[g+2]=='=' & temp[g+3]=='S' & temp[g+4]=='N') SNP=true; g++;

}

if (SNP & ref[1] == '\0' & alt[1] == '\0') {

in >> temp;

in.getline(line, 1000000);

for (int f = 0; f < 10; f++) het[h][f] = -1;

float cnts[5][2]{};

int tot[2]{};

for (int f = 0; f < 2504; f++) {

int g1 = line[f * 4 + 1] - 48;

int g2 = line[f * 4 + 3] - 48;

cnts[pops[f][1]][g1]++;

cnts[pops[f][1]][g2]++;

tot[g1]++;

tot[g2]++;

}

int pop = -1, al = -1;

for (int f = 0; f < 5; f++) {

float fq = float(cnts[f][0]) / (cnts[f][0] + cnts[f][1]);

het[h][1 + f] = fq * (1 - fq) * 2;

if (cnts[f][0] == tot[0]) pop = f, al = 0;

if (cnts[f][1] == tot[1]) pop = f, al = 1;

}

int trans = 0;

if (abs(cng(ref[0]) - cng(alt[0])) == 2) trans = 1;

if (pop > -1) het[h][6] = pop, het[h][7] = al, het[h][8] = tot[al], het[h][9] = trans;

alleles[h][0] = ref[0], alleles[h][1] = alt[0];

location[h] = loc;

if (in.eof()) location[h] = -1;

test = true;

}

else in.getline(line, 1000000);

}

}

// initialise the SNP array with 100000 values

void Load_First(ifstream& in)

{

// character arrays used to parse the vcf file fields

char temp[10000];

char ref[1000];

char alt[1000];

int cnt = 0; // SNP counter

long loc = 0;

// take 10000 SNPs from the vcf file

while (cnt < 100000) {

// load location, ref, alt and then test for whether it is described as a SNP

in >> temp >> loc >> temp >> ref >> alt >> temp >> temp >> temp;

int g = 0;

bool SNP = false;

while (temp[g] != '\0' & !SNP) {

if (temp[g]=='V' & temp[g+1]=='T' & temp[g+2]=='=' & temp[g+3]=='S' & temp[g+4]=='N') SNP=true;

g++;

}

// if it is a SNP and ref and alt are both single bases (i.e. not an indel)

if (SNP & ref[1] == '\0' & alt[1] == '\0') {

in >> temp;

// read the genotype list into ‘line’, setting recipient array to -1

in.getline(line, 1000000);

for (int f = 0; f < 10; f++) het[cnt][f] = -1;

float cnts[5][2]{};

int tot[2]{};

// extract genotype info and store region counts of both alleles, plus overall totals

for (int f = 0; f < 2504; f++) {

int g1 = line[f * 4 + 1] - 48;

int g2 = line[f * 4 + 3] - 48;

cnts[pops[f][1]][g1]++;

cnts[pops[f][1]][g2]++;

tot[g1]++;

tot[g2]++;

}

// calculate heterozygosities and test whether all minor alleles are in one region

int pop = -1, al = -1;

for (int f = 0; f < 5; f++) {

float fq = float(cnts[f][0]) / (cnts[f][0] + cnts[f][1]);

het[cnt][1 + f] = fq * (1 - fq) * 2;

if (cnts[f][0] == tot[0]) pop = f, al = 0; // all reference alleles in one region

if (cnts[f][1] == tot[1]) pop = f, al = 1; // all alternate alleles in one region

}

// test for transition / transversion

int trans = 0;

if (abs(cng(ref[0]) - cng(alt[0])) == 2) trans = 1;

// if one region has all of a minor variant, store details

if (pop > -1) het[cnt][6] = pop, het[cnt][7] = al, het[cnt][8] = tot[al], het[cnt][9] = trans;

alleles[cnt][0] = ref[0], alleles[cnt][1] = alt[0];

location[cnt] = loc;

cnt++;

}

else in.getline(line, 1000000);

}

}

// calculate the triplet value from base values for triplet (b1,b2,b3) and the new allele (b4)

int triplet(int b1, int b2, int b3, int b4)

{

{

int x = b1 * b2 * b3;

if (x == 0) return -1;

if (b2 < 3) { // if middle base of triplet = A or C

return (b4 - 1) * 32 + (b2 - 1) * 16 + (b1 - 1) * 4 + b3 - 1;

}

else { // otherwise invert the triplet bases

b1 = 5 - b1;

b2 = 5 - b2;

b3 = 5 - b3;

b4 = 5 - b4;

return (b4 - 1) * 32 + (b2 - 1) * 16 + (b1 - 1) * 4 + b3 - 1;

}

}}

// read in the list of 2504 individuals with ‘population’ and ‘region’

void LoadPops()

{

// I created a file with each 1000g individual, its population and its major region

char infile2[200] = "C:\\Users\\wa100\\Downloads\\inpops.txt";

ifstream in2(infile2);

if (!in2) cout << "inpops not open";

char line[200];

in2.getline(line, 200);

for (int i = 0; i < 2504; i++) {

in2 >> line;

in2 >> pops[i][0];

pops[i][0]--; // population numbers start at 1, c++ arrays start at zero, so decrement

in2 >> pops[i][1];

pops[i][1]--;

in2.getline(line, 200); // take sex, sample ID and endline character

}

in2.close();

}

// convert base to upper case, stripping out ‘N’s and other characters.

char convert(char base)

{

if (base == 'A' || base == 'C' || base == 'G' || base == 'T') return base;

if (base == 'a') return 'A';

else if (base == 'c') return 'C';

else if (base == 'g') return 'G';

else if (base == 't') return 'T';

return '-';

}

// read in reference bases for the current chromosome

void LoadReference(int chrom)

{

// open reference sequence file as used by the 1000g project

char infile2[200] = "B:\\hs37d5.fa";

ifstream in2(infile2);

if (!in2) cout << "not open " << infile2 << "\n";

// between each chromosome there are non-base letters, here used to count chromosomes

int cr = 0;

while (!in2.eof() && cr<chrom) { // read until focal chromosome is reached

in2.getline(line, 1000);

if (line[0] != 'A' && line[0]!='C' && line[0]!='G' && line[0]!='N' && line[0]!='T') {

cout << line << "\n";

cr++;

}

}

// read into array ‘REF’ after converting to numbers

cnt = 0;

long c = 0;

char base;

while (!in2.eof() && cnt<249999999) {

base = in2.get();

if (int(base) > 64 && int(base) < 87) {

cnt++;

c++;

if (c > 1000000) {

cout << cnt << "\n";

c = 0;

}

REF[cnt] = cng(base);

}

else if (int(base) > 89) break; // escape when lower case text detected

}

in2.close();

}

// convert base letters to numbers

int cng(char base)

{

if (base == 'A') return 1;

else if (base == 'C') return 2;

else if (base == 'G') return 3;

else if (base == 'T') return 4;

return 0;

}
